# Molecular pathology of testicular germ cell tumours: an update for practicing pathologists

**DOI:** 10.1111/his.70040

**Published:** 2025-12-12

**Authors:** Alexander Fichtner, Stefanie Zschäbitz, Daniel Nettersheim, Felix Bremmer

**Affiliations:** ^1^ Institute of Pathology University Medical Center Göttingen Göttingen Germany; ^2^ Department of Medical Oncology National Centre for Tumour Diseases (NCT), University Hospital Heidelberg Heidelberg Germany; ^3^ Department of Urology Urological Research Laboratory, Translational UroOncology, Medical Faculty and University Hospital Düsseldorf, Heinrich Heine University Düsseldorf Düsseldorf Germany; ^4^ Center for Integrated Oncology Aachen, Bonn, Cologne Düsseldorf (CIO ABCD) Aachen Germany

**Keywords:** DMRT1, germ cell neoplasia in situ, germ cell tumour, isochromosome 12p, testis

## Abstract

Testicular tumours are a diverse group of tumours, but most cases fall into the category of testicular germ cell tumours (TGCT). TGCTs are classified as either derived from a germ cell neoplasia in situ (GCNIS) or unrelated to GCNIS. Based on the development, molecular alterations and onset of development, TGCTs can further be divided into three groups. Type I TGCTs include prepubertal‐type teratoma and yolk‐sac tumour. Type II TGCTs are the only GCNIS‐related tumours in this classification and include seminomas, embryonal carcinoma, choriocarcinoma, yolk‐sac tumour and teratoma of postpubertal type. Type III TGCTs only include spermatocytic tumours. While genetic alterations are helpful in the diagnostic routine, they have not yet been useful in determining treatment options, as targetable alterations are very rare. Type I TGCTs most commonly exhibit chromosomal aberrations and rarely display alterations related to the Wnt signalling pathway. A common molecular alteration in type II TGCTs is the presence of an isochromosome 12p or gain of 12p material. It is thought that the isochromosome 12p develops during the progression of a GCNIS to an invasive TGCT. Seminomas can also exhibit c‐Kit mutations or KRAS mutations. Alterations associated with the formation of a somatic‐type malignancy and/or the development of cisplatin resistance include TP53 mutations or MDM2 gene amplifications as well as epigenetic alterations. In advanced cases, some of these genes might be useful as targeted therapies (e.g. KRAS G12C or BRAF V600E), but as these mutations are rare, studies on larger groups of patients are not possible. Amplification of chromosome 9 including the DMRT1 gene, or Ras mutations is common in spermatocytic tumours. Overlapping molecular alterations, including those on chromosome 12, have recently been discovered in some type III TGCT. Tumour serum markers (e.g. alpha‐fetoprotein, beta‐subunit of human gonadotropin and microRNAs) are helpful in the diagnosis and for follow‐up analysis to detect recurrent disease or disease progression. This review article provides an overview of the current classification of testicular tumours and their molecular classification. Furthermore, it provides information on biomarkers that are helpful in the diagnostic setting. Additionally, we will provide guidance on how to examine a testicular tumour specimen histopathologically to reach an accurate diagnosis. Finally, we will outline the importance of the content of a histopathological report for the urologists and oncologists.

## Graphical abstract

Molecular testing for isochromosome 12p is usually only necessary in difficult cases that encompass metastases or recurrent diseases including somatic‐type malignancies. So far, no breaking progress has been made in the field of targeted therapy as TGCTs only rarely show targetable molecular alterations. The necessity of new treatment options is particularly important in platin‐resistant cases created in https://BioRender.com.

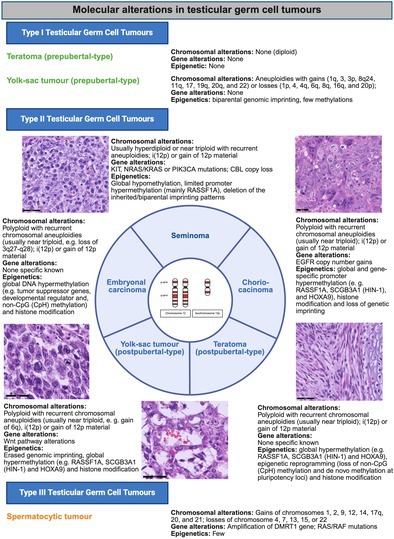

**Abbreviations**
β‐hCG: β‐subunit of human choriogonadotropin
FISH: fluorescence in situ hybridization
GCNIS: germ cell neoplasia in situ
GTS: growing teratoma syndrome
ICCR: International Collaboration of Cancer Reporting
IGCCCG: International Germ Cell Consensus Classification Group
IGF1R: insulin growth factor receptor‐1
LDH: lactate dehydrogenase
NHEJ: non‐homologous end joining
NSGCT: non‐seminomatous germ cell tumours
PLAP: placental alkaline phosphatase
SALL4: sal‐like protein 4
STM: somatic‐type malignancy
TGCT: testicular germ cell tumours

## Introduction

Primary testicular tumours most commonly affect adolescent and young adult males. According to the current WHO classification of urinary and male genital tumours, there are over 40 different histological types of testicular tumour. The most common testicular tumours belong to the group of testicular germ cell tumours (TGCT), which especially occur primarily in young adults aged 15–45, but also in children and in elderly men.^1^ Furthermore, sex cord stromal tumours, tumours of the testicular adnexa, tumours of the epididymis, and tumours of the tunica vaginalis can occur, albeit much less frequently. In addition, lymphomas may arise primarily inside the testis or manifest in the testis in cases of a systemic disease. Soft tissue tumours primarily develop in the spermatic cord but can also involve the testis. Metastases from carcinomas and other rare neoplasms must be considered in the differential diagnosis. A pathologist must keep this spectrum of tumours in mind when analysing a testicular tumour specimen.

**Figure 1:**
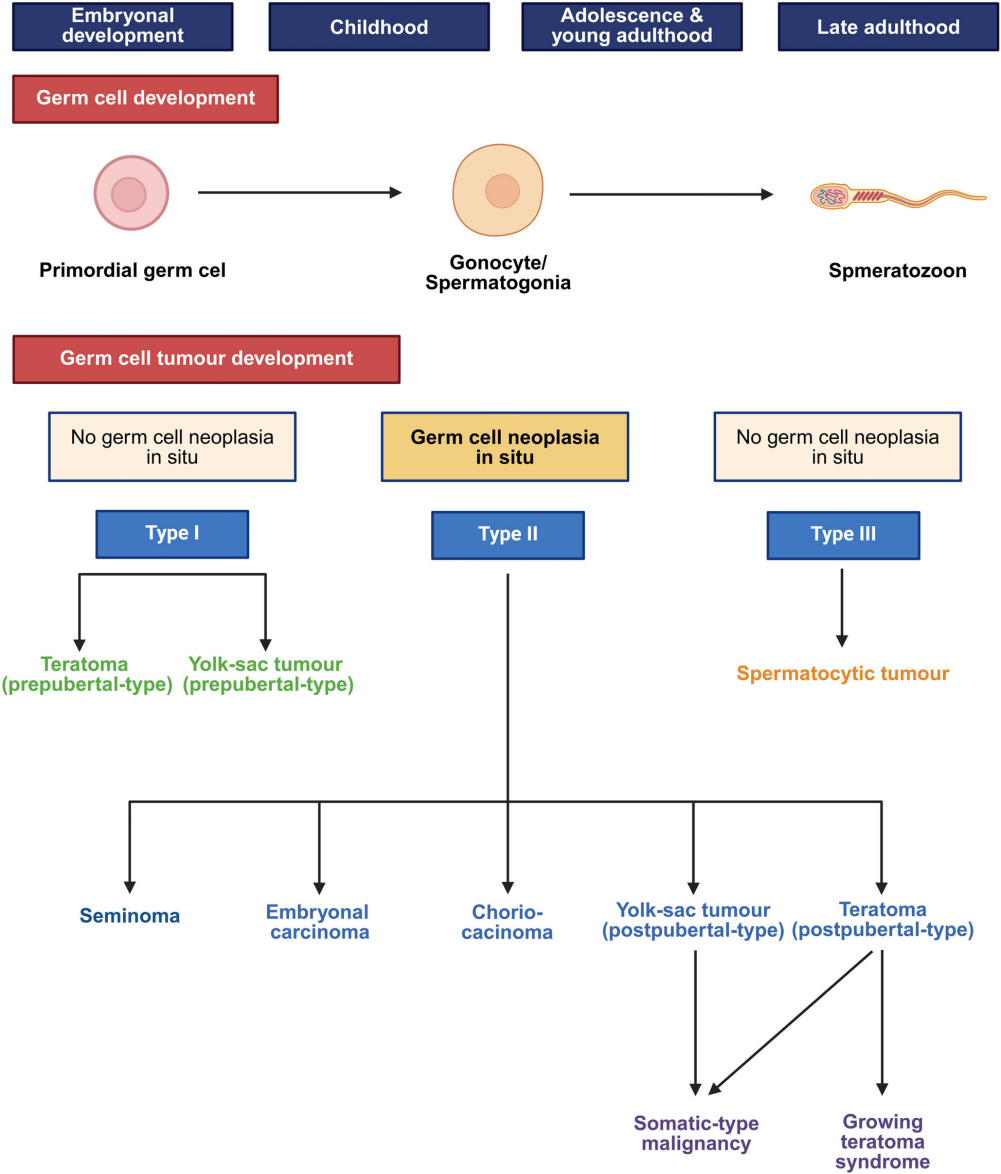
Developmental‐based classification of testicular germ cell tumours (TGCT). Germ cell tumours arise at different times of germ cells development. The primordial germ cells develop into different stages and at the stage of a gonocyte reach its niche in the testicular tumours. The gonocyte then further develops into a spermatogonia, which are the somatic stem cells for the spermatogenesis. Type I TGCT directly arise from primordial germ cells, type II TGCT develop from a common precursor lesion called germ cell neoplasia in situ (GCNIS) and type III TGCT arise from later stages, presumably at the stage of a spermatogonia. Type II TGCT, especially teratoma of postpubertal‐type and yolk‐sac tumours of postpubertal type can progress into a somatic‐type malignancy. Therapy‐resistant teratomas might grow on and lead to a so‐called growing teratoma syndrome created in https://BioRender.com.

The heterogeneous group of TGCT is classified as tumours that are derived from a germ cell neoplasia in situ (GCNIS) or unrelated to a GCNIS accordingly.^2^, ^3^ A second classification, proposed by Oosterhuis *et al*., is based on the developmental stage of the purported precursor of each tumour type. TGCTs are therefore divided into type I–III based on epidemiological, clinical, morphological and molecular differences (Figure 1).^4^, ^5^ Investigating the tumour‐adjacent testicular tubules for a GCNIS should therefore be the first step in histopathological analysis of testicular tissue. Molecular characteristics can be used to diagnose the correct TGCT subtype in the form of immunohistochemical markers or chromosomal analysis. Common alterations in type II TGCT, such as the isochromosome 12p, can be helpful in unclear cases, or when differentiating a TGCT or its metastasis from a primary somatic neoplasm. Somatic mutations are rare and targeted therapy is uncommon in the treatment of TGCT. Routine molecular testing is therefore also less common than in other solid neoplasms. Serum tumour markers can be useful in diagnosing testicular tumours and predicting histological subtypes in some cases.

## What Does the Pathologist Need to Know from the Urologist?

To diagnose a TGCT, the pathologist needs information about the serum tumour markers, which can already give a hint on the expected histological subtypes. Therefore, the urologist should provide information about these serum tumour markers to the pathologist on the application form. Furthermore, any previous cancer diagnoses should be reported, to help the pathologist with the differential diagnosis, especially in elderly men.

## Molecular Serum Biomarker Testing in Germ Cell Tumour Patients

Patients with a suspected testicular tumour should undergo serum tumour marker screening before and after the orchiectomy or chemotherapy in order to determine their risk classification according to the International Germ Cell Consensus Classification Group (IGCCCG) system.^6^ The advantages of serum tumour markers are that they can be obtained using a non‐invasive method and that testing can be repeated to monitor the disease course, reducing the need for unnecessary imaging or invasive testing.

Conventional serum tumour markers include lactate dehydrogenase (LDH), alpha‐fetoprotein (AFP) and the β‐subunit of human choriogonadotropin (β‐hCG), as well as more recently detected microRNAs. However, serum levels of LDH are neither very sensitive nor specific, as they can be elevated in different types of tumour, including all TGCT subtypes, or in cases of cell damage.^7^, ^8^ Knowledge of the levels of AFP and β‐hCG is important to make predictions about different subtypes and to later search for these subtypes in histological specimens. AFP is elevated in cases of yolk‐sac tumours,^9^ and β‐hCG is elevated in choriocarcinomas.^10^ Slight elevations of β‐hCG may be present in seminomas or NSGCTs without a visible choriocarcinoma component, as these may contain single intermingled syncytiotrophoblasts.^11^ If AFP or β‐hCG are elevated but the corresponding histology cannot be found, further tissue should be embedded and analysed.

In addition, circulating microRNAs recently detected in TGCT patients demonstrate higher sensitivity (87–96%) and specificity (99%) in seminoma and NSGCT than AFP and β‐hCG combined (about 60%), which are often negative in seminoma and teratoma.^12^, ^13^ Elevated levels of the microRNAs miR‐371a‐3p, miR‐372‐3p, miR‐373‐3p and miR‐367‐3p have been observed in the serum of patients with seminoma and NSGCT, with the exception of teratoma. Of these microRNAs, miR‐371a‐3p is the best validated and should be used in serum analysis.^12^ It enables the earlier detection of TGCTs and can be used for a more accurate monitoring of the treatment response, as well as improving surveillance for recurrence.

However, the integration of microRNAs into clinical decision‐making is not yet standard practice and prospective trials are currently underway to further investigate their role. Pathologists should play a critical role in the clinical implementation of microRNA assays, because the results must be compared with histopathological results.

## Classification of Testicular Germ Cell Tumours

### GCNIS‐Derived (Type II) Testicular Germ Cell Tumours

**Figure 2:**
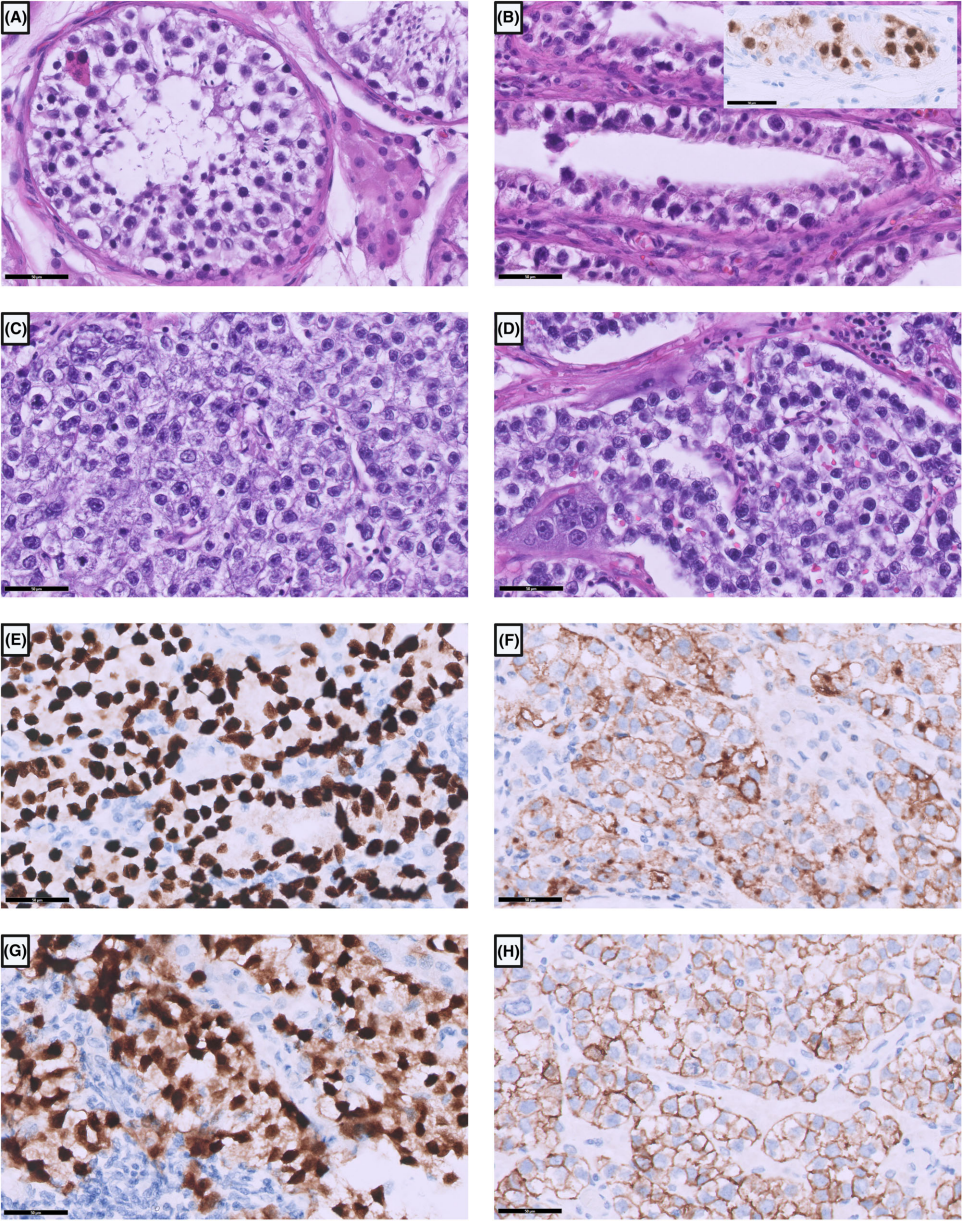
Germ cell neoplasia in situ (GCNIS) and seminoma. (**A**) Normal testicular tubule with present spermatogenesis. (**B**) Atypical germ cells without maturing spermatogenesis in case of a GCNIS with positive nuclear staining for OCT3/4 (inlet). (**C**) Seminoma consisting of atypical, low cohesive growing cells with clear cytoplasm and irregular shaped nuclei with prominent nucleoli with single intermingled syncytiotrophoblastic cells (**D**). Seminom cells stain positive for SALL4 (**E**), PLAP (**F**), OCT3/4 (**G**) and CD117 (**H**).

Type II TGCTs are the most common TGCTs and develop from reprogrammed germ cells at the stage of gonocytes, forming a GCNIS in the testicular tubules. Previous terms such as ‘carcinoma in situ’ and ‘intratubular germ cell neoplasia’ should no longer be used.^14^ This is the first feature to look for in the tumour‐adjacent testicular tubules during histopathological investigation of a testicular tumour resection specimen.^14^ Testicular tubules containing a GCNIS usually lack the typical spermatogenesis of normal testicular tissue (Figure 2A) and contain only large atypical spermatogonia‐like cells with clear to slightly eosinophilic cytoplasm and medium‐sized hyperchromatic nuclei with prominent eosinophilic nucleoli (Figure 2B). These cells are located inside the so‐called spermatogonial niche, between the basement membrane and the Sertoli cells. Other findings that can be observed in testicular dysgenesis syndrome in the non‐tumour tissue include atrophy of the testicular tubules, microcalcification or Leydig cell hyperplasia.^15^

The type II TGCT group includes seminomas and non‐seminomatous germ cell tumours (NSGCT). This distinction is important because seminomas have a far better prognosis, and different treatment options are used for seminomas and NSGCTs.^16^ NSGCTs are further subdivided into embryonal carcinomas, choriocarcinomas, yolk‐sac tumours of postpubertal type and teratomas of postpubertal type. All of these entities may also occur as components of mixed tumours.

### Histologic Subtypes of Type II Testicular Germ Cell Tumours

Morphologically, seminomas resemble atypical spermatogonia and consist of medium‐sized tumour cells with clear to slightly eosinophilic cytoplasm and medium‐sized nuclei with basophilic nucleoli (Figure 2C). The tumour cells are organized into lobules or small nests, which are divided by small collagenous septa where lymphocytic infiltrations can be found. Rarely, granulomas containing epithelioid macrophages can be found.

**Figure 3:**
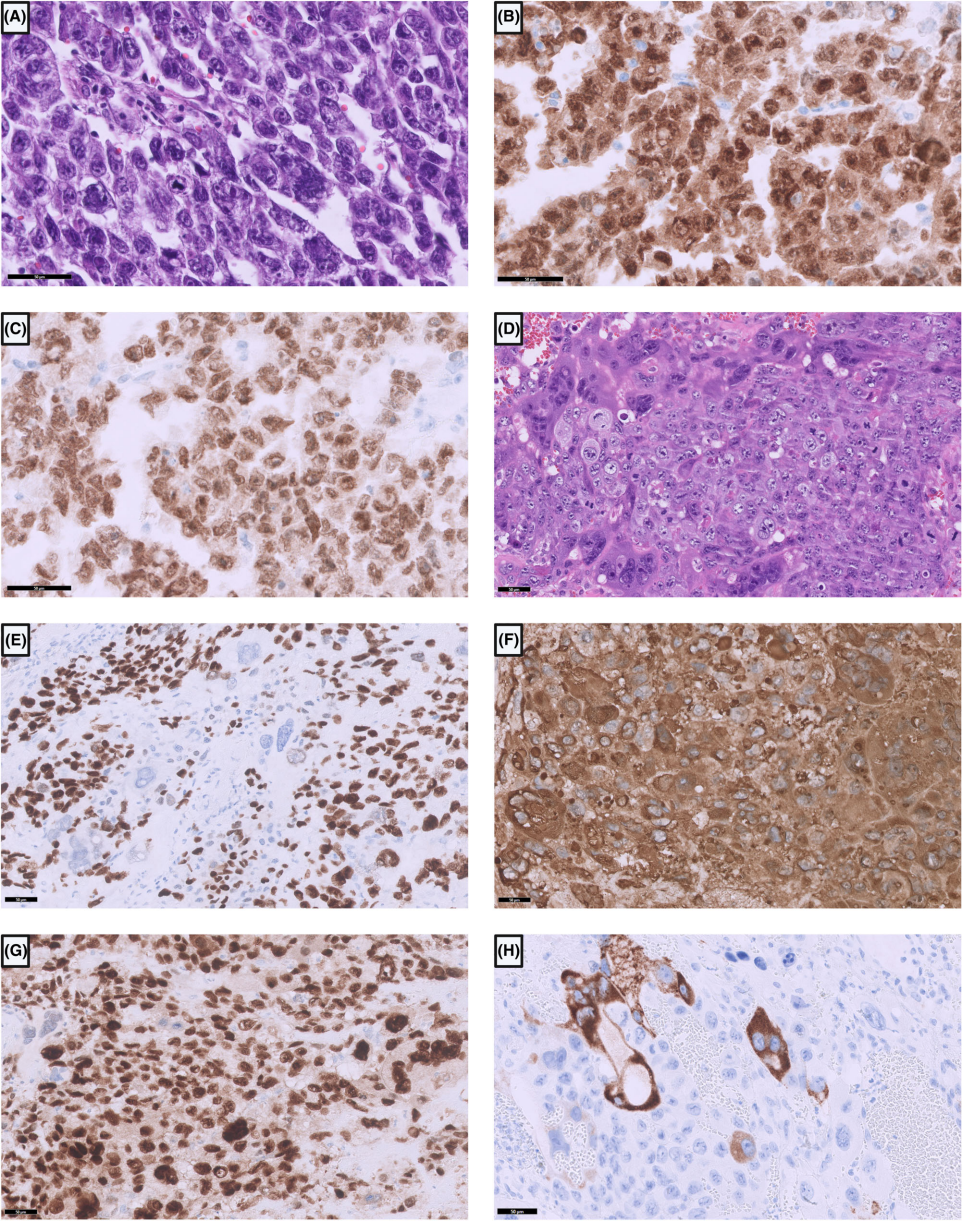
(**A**) Solid growth pattern of embryonal carcinoma consisting of polygonal pleomorphic cells with hyperchromatic nuclei and prominent nucleoli. They show positive staining for OCT3/4 (**B**) and SOX2 (**C**). (**D**) Choriocarcinomas are composed of multinucleated syncytiotrophoblasts, intermediate trophoblasts and mononuclear trophoblasts. Syncytiotrophoblasts are negative for SALL4 (**E**). All types of cells stain positive for β‐hCG (**F**) and GATA3 (**G**), but syncytiotrophoblasts stain weaker for GATA3 and are also positive for inhibin (**H**).

*Embryonal carcinomas* are the most primitive type of germ cell tumour, and their cells have hyperchromatic nuclei with prominent nucleoli, resembling embryonic stem cells (Figure 3A). The tumour cells can grow in glandular, papillary or solid patterns. Prominent necrosis and haemorrhage can be found between the tumour cells.

Choriocarcinomas consist of three different cell types, including multinucleated syncytiotrophoblasts, intermediate trophoblasts and cytotrophoblasts (Figure 3D). Prominent necrosis and haemorrhage can be found close to these tumours.

**Figure 4:**
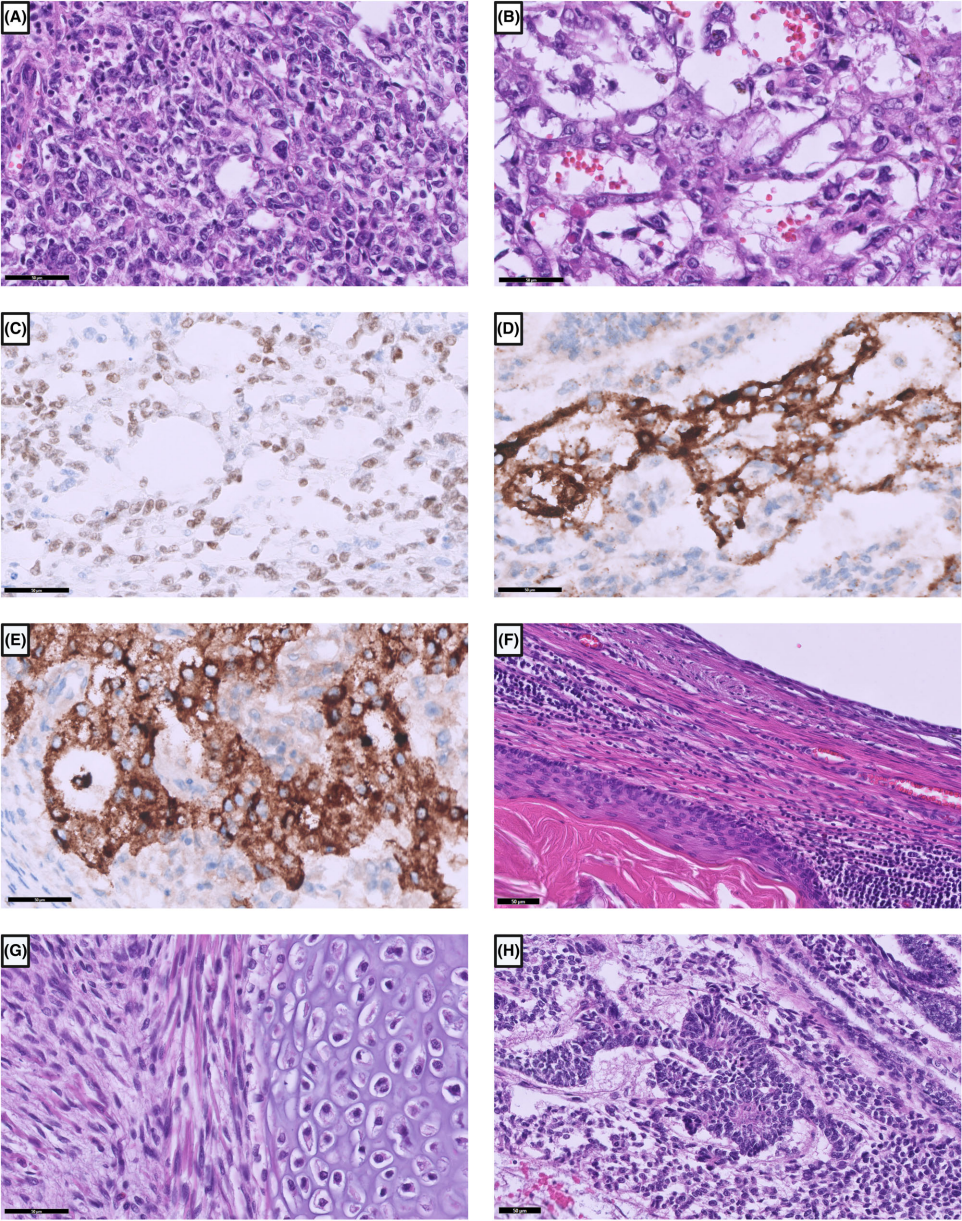
Yolk‐sac tumour and teratoma. (**A**) Yolk‐sac tumour with a solid growth pattern consisting of medium‐sized tumour cells with pleomorphic nuclei. (**B**) Microcystic to reticular growth pattern of a yolk‐sac tumour. The tumour cells stain positive for FOXA2 (**C**), Glypican‐3 (**D**) and alpha‐fetoprotein (**E**). (**F**) Mature teratoma with cystic elements lined by keratinizing squamous epithelium or flat epithelium. (**G**) Cartilage and smooth muscle cells in a teratoma. (**H**) Teratoma with immature elements of so‐called embryonic neuroectodermal tissue.

Yolk‐sac tumours of postpubertal type exhibit a high variety of growth patterns, including reticular, microcystic, papillary, glandular, hepatoid, endometrioid, solid and sarcomatoid (Figure 4A,B). In each pattern, the tumour cells have different morphologies from small cuboidal to big and polygonal. The nuclei are hyperchromatic with prominent nucleoli. The tumour cells may form a rosette‐like structure around vessels, known as Schiller‐Duval bodies.

Teratomas of postpubertal type can contain all different types of mature or immature tissue representing the three germ layers (e.g. skin and skin adnexal structures, adipose tissue, ciliated and intestinal epithelium, cartilage, bone, glial tissue) (Figure 4F–H). Differentiating between mature and immature teratomas is not relevant to their biological behaviour, as even pure mature teratomas of postpubertal type are malignant. However, immature components must be distinguished from a somatic‐type malignancy (STM).

Although TGCT growth patterns are well characterized, it should be noted that these tumours are very heterogeneous, presenting with several pitfalls and patterns. This is partly due to the possible presence of a mixture of the different subtypes in varying proportions, some of which are difficult to identify. Therefore, each histological slide must be evaluated very accurately.

One special form of TGCT is the so‐called regressed or ‘burnt‐out’ TGCT, which is particularly prevalent in patients with seminomas. In these cases, spontaneous regression can result in the primary tumour being completely replaced by scar tissue and prominent inflammation can be seen.^17^ Only a few residual vital tumour cells or GNCNIS may be present in the surrounding tubules. In these cases, the disease may be detected due to metastases, that still contain vital tumour cells. Pathologists and clinicians should be aware of this condition to avoid misclassifying metastatic TGCT as primarily extragonadal disease.^18^

In rare cases, the development of a STM is possible, in which a malignant tumour component resembling a sarcoma, carcinoma, embryonic‐type neuroectodermal tumour, glioma, nephroblastoma, haematological neoplasm or an unclassified ‘somatic‐type’ malignancy can be present (see below).^19^

### Molecular Alterations in Type II Testicular Germ Cell Tumours

Type II TGCTs commonly have chromosomal aberrations, prominent epigenetic modifications and cellular pathway alterations, though somatic mutations are relatively uncommon in comparison with other solid malignant tumours.

In around 90% of type II TGCT cases, a characteristic alteration of chromosome 12 is detected, namely an isochromosome 12p (i12p) or gains of 12p material.^20^, ^21^ These alterations are not found in GCNIS.^22^ TGCTs typically exhibit a near triploid set of chromosomes due to non‐random gains and losses, which include gains of chromosomes 7, 8 or 21 and losses of Y, 1p, 11, 13 and 18.^23^, ^24^, ^25^

Epigenetic alterations include global DNA hypomethylation in seminomas and hypermethylation in NSGCTs.^23^, ^26^ The hypermethylation in NSGCTs includes tumorsuppressor genes and developmental genes (e.g. SCGB3A1 (HIN‐1), RASSF1A and HOXA9).^27^, ^28^ Tumourigenesis and tumour progression are also promoted by histone modifications, bivalent chromatin marks and altered expression of non‐coding RNAs (e.g. microRNAs and long non‐coding RNAs).^26^, ^29^ Different pluripotency factors such as OCT3/4, SOX2 and SOX17 are differentially expressed and serve as functional drivers and diagnostic markers.^23^, ^30^

**Figure 5:**
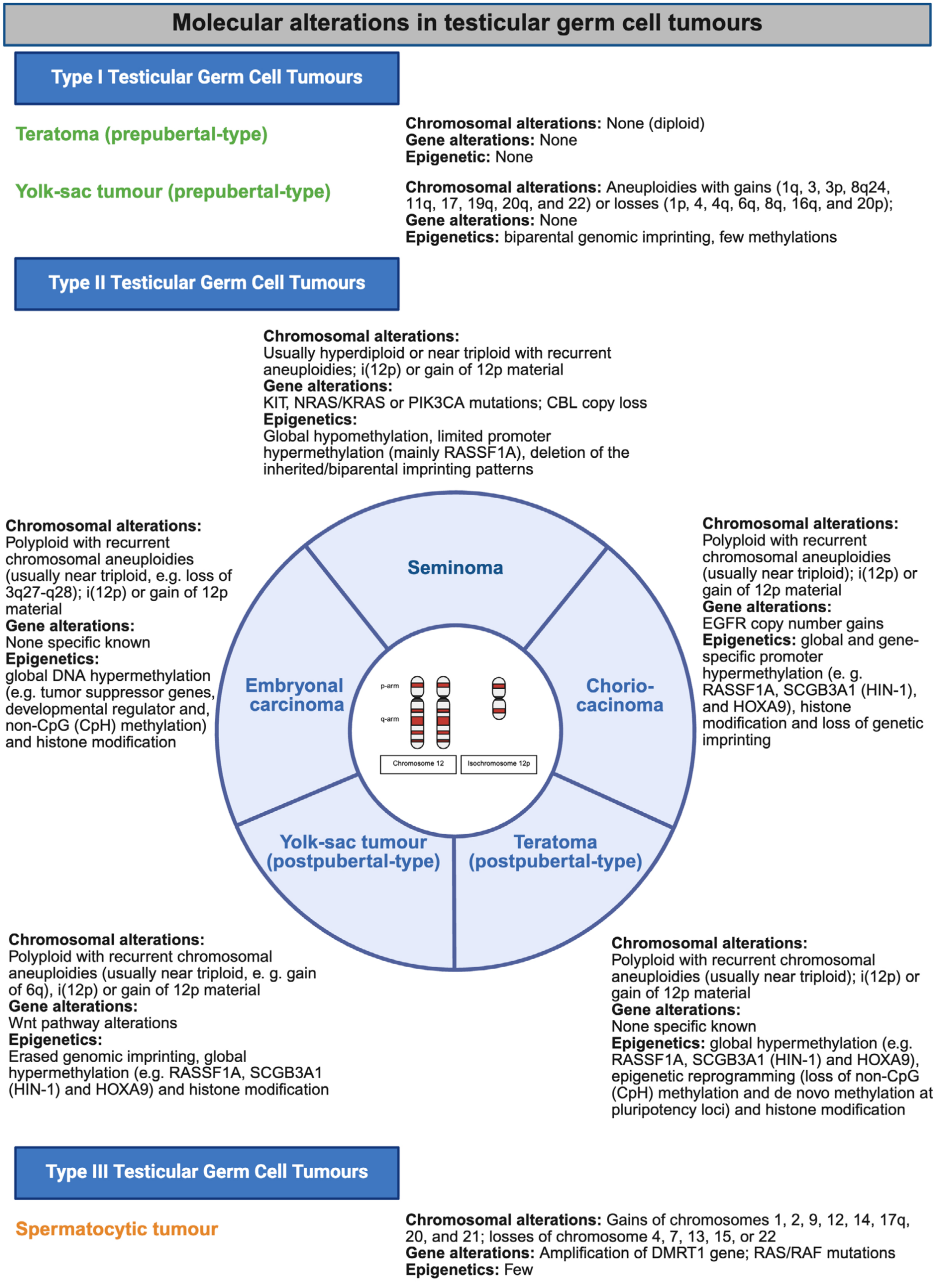
Molecular alterations of testicular germ cell tumours. The three different types of testicular germ cell tumours (TGCT) show different molecular alterations. Most common are chromosomal alterations and epigenetic alterations. Type II TGCT share alterations of chromosome 12, namely the isochromosome 12p (i(12p)) or gains of chromosomal 12. Type III TGCT harbour a characteristic amplification of *DMRT1* gene created in https://BioRender.com.

Other genetic alterations in type II TGCT include the KIT signalling, TP53 pathway, the WNT signalling and other rare pathways. Alterations to the KIT‐KITLG pathway are often found in seminomas (up to 25%) and in mixed NSGCT with a seminoma component.^23^ GCNIS cells do not show any mutations in the KIT‐KITLG pathway, except in cases where they are in direct proximity to invasive TGCT.^31^ KRAS and NRAS are genes involved in pathways downstream from KIT, and mutations in these genes promote cell proliferation and tumour development. BRAF mutations, on the other hand, are very rare. KRAS G12C and BRAF V600E mutations, in particular, might be possible targets for molecularly targeted therapy. However, large studies to implement therapy are not possible to perform. In some cases, mutations in the *TP53* gene or degradation of the TP53 protein caused by *MDM2* gene amplification, can be detected and are associated with cisplatin resistance.^32^, ^33^ Recently, XU and colleagues detected different WNT signalling activation mechanisms in type II TGCTs as well as type I TGCTs. This leads to proliferation, migration and differentiation, which is associated with a worse outcome than WNT‐wild‐type tumours.^34^ Loss of 3q27‐q28 was detected in embryonal carcinoma,^35^ as were mutations of *B3GNT8*, *CAPN7*, *FAT4*, *GRK1*, *TACC2* and *TRAM1L1*.^36^ Choriocarcinomas show either an amplification, a higher copy number or an overexpression of *EGFR* (Figure 5).^37^ Unfortunately, these alterations have not yet been a successful target in the therapy of TGCTs and have not been very useful clinically.

## GCNIS‐Unrelated Germ Cell Tumours

### Type I Testicular Germ Cell Tumours

Type I TGCTs most often develop in young children and arise directly from primordial germ cells that have undergone a maturation arrest without the formation of a visible precursor lesion. This is why they are often referred to as TGCTs of prepubertal type.^4^ Type I TGCTs include teratomas and yolk‐sac tumours of prepubertal type and mixed tumours. These teratomas are composed of organoid structures including epidermoid or dermoid cysts, that can also occur in older men.^38^ They usually have a benign biological behaviour, with rare metastasizing cases reported only in a few cases of immature teratomas or neuroendocrine tumours of prepubertal type.^39^ Conversely, yolk‐sac tumours are malignant neoplasms that can metastasize.^40^ Histomorphologically, all growth patterns observed in yolk‐sac tumours of postpubertal type can be found in type I yolk‐sac tumours, except for a sarcomatoid growth pattern, which has not been reported so far.

Type I tumours only show chromosomal aberrations (gains and losses), but no other notable molecular alterations. Type I teratomas usually lack chromosomal aberrations and type I yolk‐sac tumours can exhibit multiple aneuploidies with gains (1q, 3, 3p, 8q24, 11q, 17, 19q, 20q and 22) or losses (1p, 4, 4q, 6q, 8q, 16q and 20p) of various chromosomes (Figure 5).^41^, ^42^, ^43^ Moreover, type I TGCTs can have epigenetic changes. These TGCTs have a low mutational burden, and it has only recently been shown that alterations in the WNT signalling pathway in type I yolk‐sac tumours were shown to be caused by mutations, copy number alterations or promoter methylation.^34^ Other frequently methylated genes include H19, IG2, SNRPN and RUNX3.^44^, ^45^, ^46^

## Type III Germ Cell Tumours

**Figure 6:**
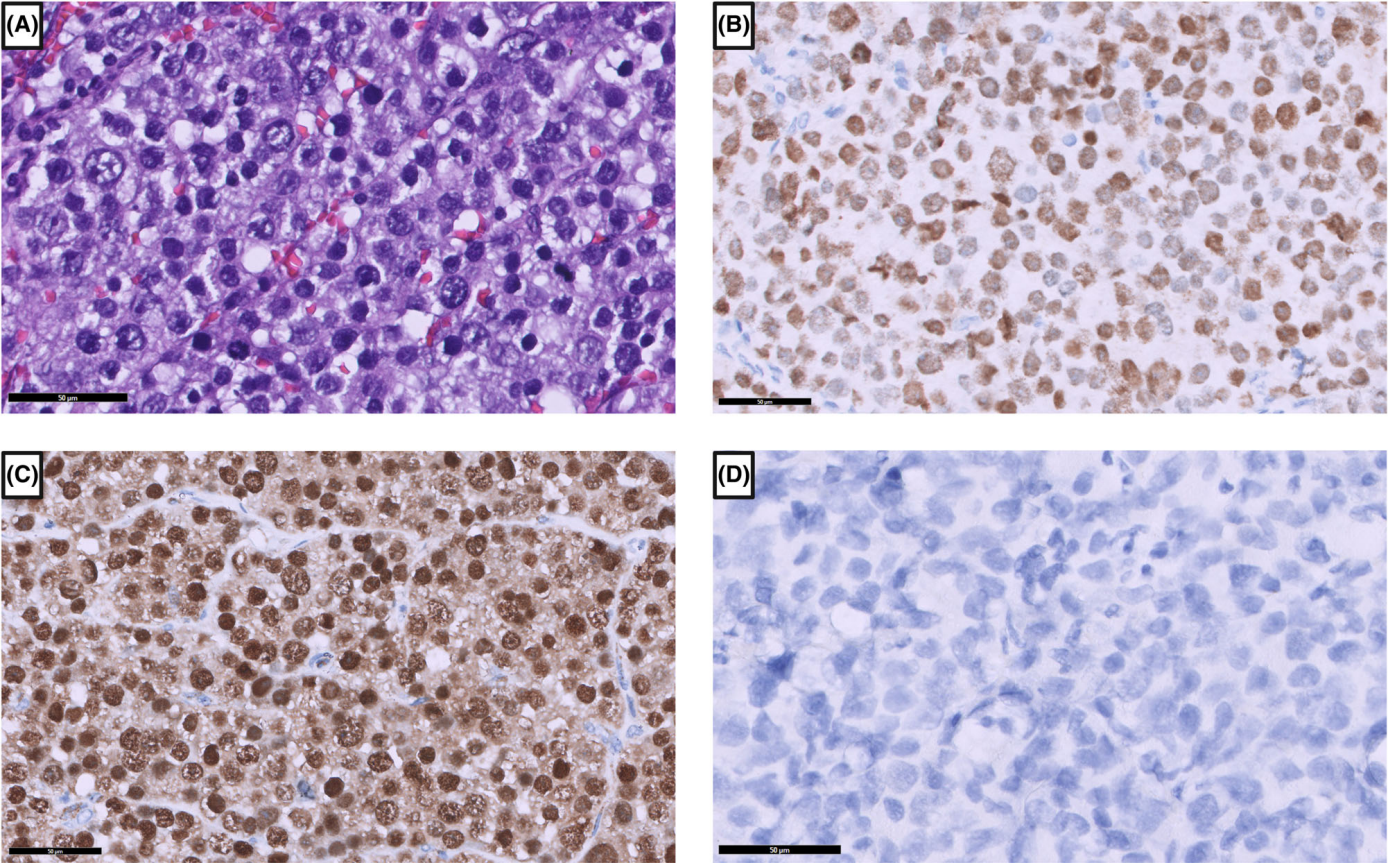
Spermatocytic tumour. (**A**) Spermatocytic tumours are composed of three different cell populations including large, medium and small sized tumour cells with different chromatin patterns. These cells stain positive for SALL4 (**B**), and positive for DMRT1 (**C**), but negative for OCT3/4 (**D**).

Type III TGCTs only include the so‐called spermatocytic tumour, which was previously known as spermatocytic seminoma. This tumour was reclassified because of different morphological and molecular characteristics in comparison to seminomas. It typically consists of three different populations of tumour cells that are arranged in solid nests (Figure 6A). Spermatocytic tumours usually occur in elderly men between the age of 50 and 60, but can arise in men of all ages (19–92 years).^47^, ^48^, ^49^ These tumours may have gains in chromosomes 1, 2, 9, 12, 14, 17q, 20 and 21, as well as losses in chromosomes 4, 7, 13, 15 or 22. The most characteristic change is an amplification of chromosome 9 including the *DMRT1* gene (Figure 5).^50^, ^51^

Frequent molecular alterations in spermatocytic tumours identified by Gupta *et al*. revealed that there is one group with global ploidy shifts without recurrent mutations and a second group with diploid genomes and RAS/RAF mutations. A malignant biological behaviour with a possible metastasizing disease course is found in anaplastic or sarcomatoid tumours, which may be associated with relative gains of 12p and/ or mutations of *TP53*.^51^ These findings, along with the presence of overlapping methylation profiles and microRNA‐371/−372/−373 expression levels, suggest that some spermatocytic tumours share features with non‐teratomatous type II TGCTs.^52^, ^53^

## Molecular Immunohistochemical Markers for Differential Diagnosis

It is important to have a basic knowledge of the use of immunohistochemical markers in the diagnosis of TGCT, and to be aware of potential pitfalls, especially at metastatic sites. In general, a marker panel should be established that can be used according to the suspected subtypes. Sal‐like protein 4 (SALL4) is a marker that is positive in non‐neoplastic germ cells, GCNIS, as well as type I to III TGCTs and is therefore referred to as a TGCT origin marker because it is physiologically important during embryonal development.^54^ LIN28A can also be used but it is less frequently positive and its cytoplasmic staining is more difficult to analyse than the nuclear staining of SALL4.^55^ Placental alkaline phosphatase (PLAP) has been used as well as a germ cell marker but is less sensitive than SALL4.^56^

GCNIS cells stain positively for OCT3/4 (Figure 2B, insert), a transcription factor that is physiologically present in spermatogonia from birth until the first year of life, but which is aberrantly expressed in GCNIS.^57^ Other positive markers include PLAP, D2‐40 and CD117.^58^

The invasive tumour components can be differentiated from each other using different markers. OCT3/4 is a useful marker to stain because it is only positive in seminomas (Figure 2G) and embryonal carcinomas (Figure 3B), but negative in the other three subtypes of type II TGCT and also negative in spermatocytic tumours.^59^ NANOG is another marker that can be used to detect GCNIS, seminoma and embryonal carcinoma. However, OCT3/4 has superior specificity, a robust nuclear staining and has been used in a wide range of diagnostic settings.^59^, ^60^ Seminomas are also positive for D2‐40, SOX17 and CD117 (Figure 3H), while embryonal carcinomas are positive for SOX2 (Figure 3C) and CD30.^61^, ^62^ In choriocarcinomas, mononucleated trophoblasts stain positive for SALL‐4 (Figure 3E) and p63. All three cell types stain positively for β‐hCG (Figure 3F) and GATA3 (Figure 3G), the latter of which can also be positive in yolk‐sac tumours.^63^ Syncytiotrophoblasts are also positive for inhibin (Figure 3H). Yolk‐sac tumours are positive for FOXA2, Glypican‐3 and AFP (Figure 4E), of which AFP has the lowest sensitivity, as well as HNF1‐β, SOX17, and CDX2.^64^, ^65^ Teratomas can be positive for almost all the above‐mentioned markers with weak and only focal positive stainings, except for OCT3/4, which is usually negative. Therefore, the use of immunohistochemical markers in teratomas is not very useful.

Spermatocytic tumours are positive for SALL4 (Figure 6B), OCT2, MAGEA4, KIT, SSX2, SYCP1, XPA and SAGE1, as well as DMRT1 (Figure 6C) and negative for OCT3/4 (Figure 6D), PLAP, FOXA2, AFP and β‐hCG.^50^, ^66^ The combination of OCT3/4 (−), CD117 (+) and DMRT1 (+) is sufficient to confirm cases of unclear spermatocytic tumours and differentiate them from type II TGCTs.

## Molecular Fundamentals of Somatic‐Type Malignancy

A type II TGCT can develop into a STM. This is a germ cell tumour that not only includes one or more of the conventional histological subtypes, but also malignant components that resemble a somatic neoplasm, such as sarcoma (e.g. rhabdomyosarcoma and angiosarcoma), carcinoma (often adenocarcinomas), embryonic‐type neuroectodermal tumour, glioma, haematological neoplasm and others.^67^

According to the current WHO classification, a diagnosis of a STM should be made, if such a tumour component accounts for at least ≥5 mm.^68^ Immunohistochemically, these tumours might still harbour positive staining for TGCT markers such as SALL4, but they are particularly positive for markers typically found in the somatic counterpart (e.g. muscle markers in rhabdomyosarcoma).

Historically, most cases of STM were found in tumours containing a teratoma component. Recent studies have shown that some subsets of STMs, especially carcinomatous components, may develop from a yolk‐sac tumour component.^19^, ^69^ TGCTs with STM show aneuploidy and complex copy number changes, including focal deletions and amplifications (e.g. MDM2) as well as mutations in KRAS and TP53 genes.^70^, ^71^ Most significantly, the development of STM from a conventional TGCT is characterized by changes in DNA methylation patterns, such as increased promoter hypermethylation and altered methylation of genes involved in cell cycle regulation and different signalling pathways (e.g. MAPK/RAS and WNT).^19^, ^69^ Therapy resistance mechanisms that were found include methylation‐driven silencing of genes that impair apoptotic responses and promote survival pathways.^19^ Potential biomarkers and therapeutic targets (e.g. FGF signalling and DNA repair) have been identified in methylation analysis of specific CpG sites and promoter regions.^69^

## Molecular Aspects of Growing Teratoma Syndrome

Growing teratoma syndrome (GTS) describes cases of mixed TGCT that have metastasized, in which the teratoma component continues to grow because of chemotherapy resistance despite a decline in serum tumour markers.^72^ Because of the resistance, surgery remains the only treatment option. Molecular analysis of GTS tumours by Mego *et al*. revealed a dysregulation of the MAPK and PI3K‐Akt signalling pathways and a differential expression of 14 genes in GTS compared to non‐growing teratomas. Pongratanakul *et al*. demonstrated that there are both slow‐ and fast‐growing cases of GTS.^73^ Their proteomic and epigenetic analyses also revealed that GTS tumours are enriched in proteins involved in cell cycle regulation, cell proliferation and DNA replication. In comparison to non‐growing teratomas, the GTS also shows altered DNA methylation and microRNA expression profiles. Furthermore, they hypothesised that some cases of GTS may show an expression of pluripotency‐ and yolk‐sac tumour‐associated genes which could indicate that there is a non‐visible NSGCT component that might regrow over time if the lesion is not completely removed. This information implies that the original definition of a GTS by Logothetis needs to be updated. Pongratanakul *et al*. therefore suggested that a GTS describes a continuously growing teratoma that may still contain occult NSGCT components, which could outgrow over time.

## Molecular Mechanisms of Therapy Resistance

The favourable prognosis of TGCT, even in advanced stages of the disease (e.g. metastases), is related to the treatment with chemotherapy regimens that include cisplatin.^74^ Only 5%–10% of TGCT patients, especially those with a NSGCT, develop cisplatin resistance.^75^ The development of cisplatin resistance is a multifactorial event involving genetic, epigenetic and cellular pathway alterations that can coexist.

Pathway alterations in cisplatin resistance include alterations in apoptotic pathways. TP53 mutations or, more commonly, MDM2 amplifications have therefore been identified as independent risk factors for TGCT prognosis.^33^, ^76^ Elevated MDM2 levels promote p53 ubiquitination and degradation, recruit transcriptional co‐repressors of p53 and reduce apoptosis. In resistant TGCT cell lines the MDM2/p53 complex is more stable, which requires higher cisplatin doses to achieve cell death. Intrinsic apoptotic pathways in TGCT also involve the FAS receptor, BAX, PUMA and NOXA. Formation of a complex that consists of NOXA/MCL‐1/CDK2 is a critical component of the vulnerability towards cisplatin. Higher NOXA levels have been found to be associated with more favourable disease outcomes of TGCT.^77^ In patients with sensitive disease, cisplatin induces up‐regulation of NOXA. Binding of NOXA to the MCL‐1/CDK2 complex enhances the phosphorylation, and hence, the degradation of the proapoptotic protein MCL‐1 by CDK2. Inhibiting CDK2 with the cytoplasmic CDK inhibitor p21 or mutations of MCL‐1 may disturb this process and thus reduce cell death.^78^

Other possible mechanisms of cisplatin resistance include mutations or epigenetic changes in the PIK3CA, AKT, RAS and FGFR3 genes.^79^, ^80^ The PDGFR/PI3K/Akt signalling pathway is often overactivated in cisplatin‐resistant tumours, leading to the relocation of p21 to the cytoplasm, phosphorylation of p21 and MDM2. High expression of insulin growth factor receptor‐1 (IGF1R) has been found in TGCT cell lines with acquired cisplatin resistance.^81^ IGFR1 up‐regulation promotes overexpression of AKT and phospho‐AKT. IGFR1 silencing by long‐term shRNA has been shown to restore cisplatin sensitivity in vitro.

Furthermore, gains of chromosome 3p25.3 are also associated with cisplatin resistance and can be used as an independent risk factor for the outcome of TGCTs.^82^ In addition, cisplatin‐resistant TGCTs change their DNA repair mechanisms. These include a shift from non‐homologous end joining (NHEJ) to homologous recombination repair, which leads to a reduction of cisplatin‐induced cytotoxicity.^83^ Another cause of cisplatin resistance is epigenetic alteration, including global DNA hypermethylation (e.g. tumour suppressor gene promoters) and chromatin remodelling.^84^

## Molecular Analyses in Everyday Diagnostic Practice and for Treatment Options

Molecular analyses are rarely used in the daily diagnosis of TGCTs as most cases can be solved using morphological features or immunohistochemically staining patterns. Molecular analyses include testing the formalin‐fixed, paraffin embedded tumour tissue for isochromosome 12p or gain of 12p material. This can be performed via fluorescence in situ hybridization (FISH) or quantitative real‐time polymerase chain reaction.^21^ These analyses are necessary for primary testicular tumours or metastases with an uncommon morphological appearance, somatic‐type malignancies or to exclude a GCT in a non‐GCT tumour. For type III TGCTs, molecular alterations of chromosome 9 can be analysed by FISH, which is not necessary as these tumours show a characteristic immunohistochemical profile including positivity for DMRT1.

So far, there are no frequent gene mutations that are therapeutically targetable in germ cell tumours; therefore, routine testing is not recommended. Selected cases in treatment‐resistant cases might be analysed for a molecular tumour board review.

## What Does the Oncologist Need to Know in the Pathological Report?

A structured survey should be used for the pathological report. This should include the correct histological subtype of the TGCT and, in case of a mixed TGCT, the percentage of each subtype. Furthermore, the tumour's correct histopathological stage, information about the invasion of blood and lymph vessels as well as nerve sheath invasion, and the resection status should be reported. Some of this information has prognostic significance, for example tumour size and infiltration of the rete testis for seminomas or the presence of lymph vessel invasion or embryonal carcinoma as a histologic subtype in NSGCTs. Nowadays, the International Collaboration of Cancer Reporting (ICCR) provides a structured reporting system for a lot of different tumour types that should be used internationally. This is also described in a separate article by Bremmer and Berney in the same issue of *Histopathology*.

## Conclusion

Correct TGCT diagnosis and subtyping are necessary for choosing the right treatment option and for predicting the prognosis. Therefore, the right amount of clinical information especially regarding the knowledge of serum tumour biomarkers is important for pathologists to know. Morphological analysis is the key standard for TGCT subtyping, which can be supported by immunohistochemical analysis. Immunohistochemical markers can be used additionally to detect small subsets or to verify uncommon growth patterns of a TGCT subtype. Molecular testing for isochromosome 12p is usually only necessary in difficult cases that encompass metastases or recurrent diseases including somatic‐type malignancies. So far, no breaking progress has been made in the field of targeted therapy as TGCTs only rarely show targetable molecular alterations. The necessity of new treatment options is important in platin‐resistant cases. Selected cases of treatment resistant patients should be selected for broader molecular investigations in the setting of a molecular tumour board.

## Author contributions

FB and AF wrote the initial draft manuscript with final review and revision by DN and SZ. AF produced the figures.

## Funding information

None.

## Conflict of interest

The authors confirm that there are neither conflicts of interest nor competing interests.

## Data Availability

The data that support the findings of this study are available from the corresponding author upon reasonable request.
